# Excited-State Dynamics of CS_2_ Studied by Photoelectron Imaging with a Time Resolution of 22 fs

**DOI:** 10.1002/asia.201100458

**Published:** 2011-10-13

**Authors:** Takao Fuji, Yoshi-Ichi Suzuki, Takuya Horio, Toshinori Suzuki

**Affiliations:** a CREST, Japan Science and Technology AgencySanbancho, Chiyoda-ku, Tokyo 102-0075 (Japan), Fax: (+81) 75-753-3974 E-mail: suzuki@kuchem.kyoto-u.ac.jp; b Advanced Science InstituteRIKEN, 2-1 Hirosawa, Wako 351-0198 (Japan); c Department of Chemistry, Graduate School of Science, Kyoto UniversityKyoto 606-8502 (Japan)

**Keywords:** carbon disulfide, photochemistry, photoelectron spectroscopy, photoionization, wave packet dynamics

## Abstract

The ultrafast dynamics of CS_2_ in the ^1^B_2_(^1^Σ_u_^+^) state was studied by photoelectron imaging with a time resolution of 22 fs. The photoelectron signal intensity exhibited clear vibrational quantum beats due to wave packet motion. The signal intensity decayed with a lifetime of about 400 fs. This decay was preceded by a lag of around 30 fs, which was considered to correspond to the time for a vibrational wave packet to propagate from the Franck–Condon region to the region where predissociation occurred. The photoelectron angular distribution remained constant when the pump–probe delay time was varied. Consequently, variation of the electronic character caused by the vibrational wave packet motion was not identified within the accuracy of our measurements.

## Introduction

**Figure 1:**
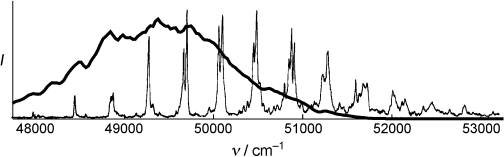
Absorption spectrum of CS_2_ (—) at a low temperature overlaid with the spectrum of the pump laser used in the present study (**—**). The absorption spectrum was adapted from Ref. [1] and reprinted with permission from R. J. Hemley et al., *J. Chem. Phys. 79*, 5219. Copyright 1983, American Institute of Physics.

The absorption spectrum of jet-cooled carbon disulfide (CS_2_) in the wavelength region of 208–192 nm (see Figure 1) exhibits distinct vibrational structures due to symmetric stretching (*ν*_1_′=392 cm^−1^) and bending (*ν*_2_′=426 cm^−1^) modes.[1] The excited electronic state responsible for this absorption band is ^1^B_2_(^1^Σ_u_^+^), which has a bent equilibrium geometry (the S–C–S bond angle is 153°[2]). Although ^1^B_2_(^1^Σ_u_^+^) adiabatically correlates to CS(X^1^Σ^+^)+S(^1^S) and is strongly bound in a linear geometry, it undergoes avoided crossing with the repulsive ^1^B_2_(^1^Π_g_) state in the bent geometry and induces predissociation to CS(X)+S(^1^D). Predissociation also occurs to CS(X)+S(^3^P) and the branching ratio for this channel is larger than that for the singlet channel: S(^3^P_J_)/S(^1^D) is 2–3.[3–5]

Farmanara et al. performed (1+1′) pump–probe photoionization mass spectrometry and found that the lifetime decreased from 620 to 180 fs when the pump wavelength was reduced from 207 to 194 nm.[6] Interestingly, the lifetime remained almost constant near the center of the above wavelength range, where the barrier to linearity of the ^1^B_2_(^1^Σ_u_^+^) state (between 48 500 and 51 000 cm^−1^) has been predicted. Because *hν*_1_′(*a*_1_) and *hν*_2_′(*a*_1_) have similar vibrational energies, a femtosecond pump pulse coherently excited adjacent vibronic bands separated by 24–56 cm^−1^, thereby generating vibrational quantum beats.[6]

Townsend et al.[7] performed (1+1′) pump–probe photoelectron spectroscopy and reproduced the vibrational quantum beats observed by Farmanara et al. They suggested that the time-dependent photoelectron kinetic energy distribution (PKED) consists primarily of two components with short (<50 fs) and long (≈500 fs) lifetimes; these lifetimes have a slight dependence on the polarization directions of the pump and probe laser pulses. This double exponential decay was reproduced by Bisgaard et al.[8] who measured *τ*_1_=(70±20) and *τ*_2_=(830±40) fs by using pump and probe pulses with wavelengths of 201.2 and 268.3 nm, respectively. They analyzed the photoionization signal intensity and the photoelectron angular distribution (PAD) for photoionization to a single vibronic state (0,0,0) of CS_2_^+^.[8] The photoelectron intensity of this selected ionization channel exhibited a vibrational quantum beat with a time period of (1010±20) fs, while PAD exhibited a time dependence with a period of about 800 fs. Photoionization to the zero vibrational state of the cation limited their observation window to a narrow geometrical region lying within the amplitude of the zero-point vibration of CS_2_^+^. Therefore, their finding implies that the character of the ^1^B_2_(^1^Σ_u_^+^) electronic wave function varies within this width.

Herein, we report time-resolved photoelectron imaging (TRPEI) of CS_2_ with an unprecedentedly high time resolution of 22 fs. The 17 fs pump pulse creates a vibrational wave packet on ^1^B_2_ and a 14 fs probe pulse interrogates its time evolution by observing PKED and PAD. As Figure 1 shows, the pump pulse coherently excites many vibronic bands and creates a spatially confined vibrational wave packet on ^1^B_2_. When a Gaussian wave function of the (0,0,0) state in X (^1^Σ_g_^+^) is projected onto ^1^B_2_ in the short pulse limit, the full-width at half maximum (FWHM) of the bending wave function is about 7°, which is considerably smaller than the bending angle of the equilibrium geometry of the ^1^B_2_ state (13°=(180°−153°)/2).

## Results

In (1+1′) resonance-enhanced multiphoton ionization with parallel polarization vectors of the pump and probe pulses, the time-dependent photoionization differential cross-section, *I*(*t*, *E*, *θ*), can be expressed by Equation (1):


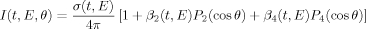
(1)

in which *E* is the photoelectron kinetic energy (PKE), *θ* is the angle between the photoelectron momentum and the laser polarization direction, and *P*_n_(*x*) is the *n*th order Legendre polynomial. Integrating Equation (1) over *E* and *θ* yields the time-dependent photoionization signal, *I*(*t*), which is proportional to the total photoelectron intensity observed as a function of the delay time, *t*. Integrating Equation (1) over only *θ* yields the time-dependent PKED, σ(*t, E*).

**Figure 2:**
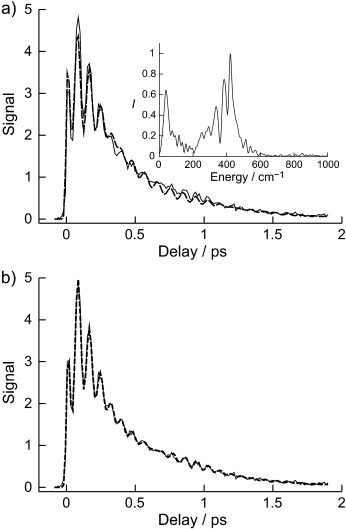
a) Observed time profile of photoionization signal intensity (—) and fitted curve using five functions (- - - -). The inset shows the power spectrum of the photoionization signal. b) Observed time profile of photoionization signal intensity (—) and a fitted curve using six functions (- - - -).

Figure 2 a shows the observed time profile of the photoelectron signal intensity (solid line). The intensity decays rapidly within 1 ps, in agreement with previous experimental results.[6–8] However, unlike previous results, it exhibits strong and rapid oscillations. The inset of Figure 2 a shows the Fourier transform of the time profile for *t*=0.1–2 ps, where the abscissa is the energy-level spacing in units of inverse centimeters, which was calculated from the beat frequency. It exhibits four strong signals at 40, 338, 386, and 424 cm^−1^ (with uncertainties of ±17 cm^−1^). The two highest values are, respectively, the vibrational level spacings of the symmetric stretching mode, *hν*_1_′ (392 cm^−1^), and the bending mode, *hν*_2_′ (426 cm^−1^), in the energy range 49 000–51 000 cm^−1^ (Figure 1).[1] The 338 cm^−1^ component corresponds to *h*(2*ν*_1_′−*ν*_2_′). The lowest energy component, 40 cm^−1^, is the difference in the energies of *hν*_1_′ and *hν*_2_′, which has been observed previously.[6–8]

Figure 2 a also shows the simulated time profile (dashed line) for single exponential decay (exp(−*t*/*τ*_1_)) with damped oscillations (exp(−*t*/*τ_n_*)cos(2π*f_n_t*+*φ_n_*), *n*=2, 3, 4, 5). It agrees reasonably well with the observed profile. Even better agreement can be obtained when an induction time *τ*′ (≈30 fs) prior to the decay is assumed (see Figure 2 b). We observed similar induction times in TRPEI of benzene and toluene in previous studies.[9] The ^1^B_2_(^1^Σ_u_^+^) state interacts with other electronic states with bent geometries to undergo predissociation to form CS(X^1^Σ^+^) and the ^1^D or ^3^P states of atomic sulfur.[10] The observed induction time is interpreted as the time for the wave packet to move from the Franck–Condon region to the critical geometry for predissociation.

**Figure 3:**
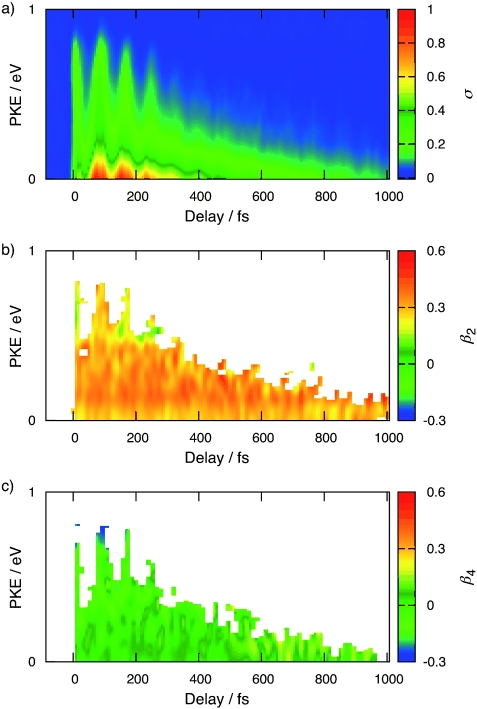
Time–energy maps of a) photoelectron intensity and anisotropy parameters b) *β*_2_ and c) *β*_4_. Only data points for *β_n_* with a standard error of the mean smaller than 0.1 are shown.

**Figure 4:**
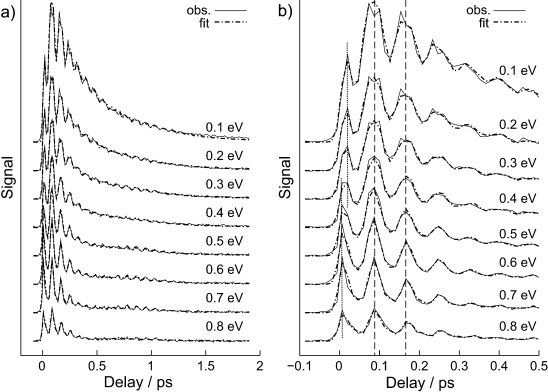
a) Time evolutions of photoelectron intensities at selected PKE subsections of carbon disulfide and b) time evolutions with expanded timescales around time zero. The dotted lines indicate the initial peaks, whereas the dashed lines indicate the second and third peaks.

**Figure 5:**
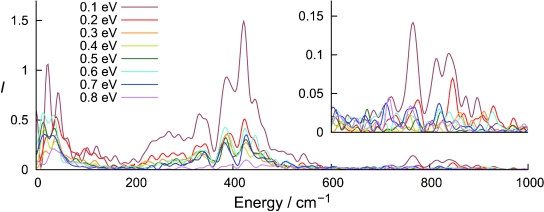
Fourier transforms of energy-resolved photoelectron signals. The inset shows an expanded view for the energy region from 600 to 1000 cm^−1^.

**Figure 6:**
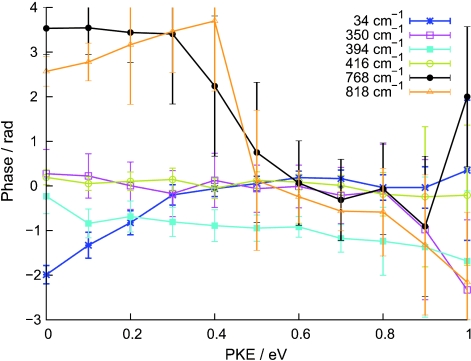
Initial phases of oscillatory components determined by fitting.

Figure 3 shows time–energy maps of the photoelectron intensities, *σ*(*t*,*E*) also shown as a 3D plot in Figure S1 in the Supporting Information, and angular anisotropies, *β_n_*(*t*,*E*). Time profiles of the photoelectron intensities at different PKEs were extracted from Figure 3 and are presented in Figure 4. The fitted curves incorporated the above-mentioned induction time. Close examination of the oscillatory features in Figure 4 b reveals that the peaks occur with the same time delay (as indicated by broken lines); however, the peaks are systematically sharper at higher PKE. Note also that the time of the initial peak exhibits a systematic shift. These observations indicate that there are overtones (790–850 cm^−1^) of the main beating component (386 or 424 cm^−1^). Figure 5 shows the Fourier transforms of the profiles shown in Figure 4. The overtone components in Figure 5 are clearer than those shown in the inset of Figure 2 a. The (initial) phases of the overtones are about π at low PKE and are zero at high PKE (see Figure 6). Mathematically, the variation in the peak shape is caused by the phase difference between the 800 and 400 cm^−1^ components; however, it ultimately originates from two wave packets moving toward and away from a single classical turning point, as discussed below.

**Figure 7:**
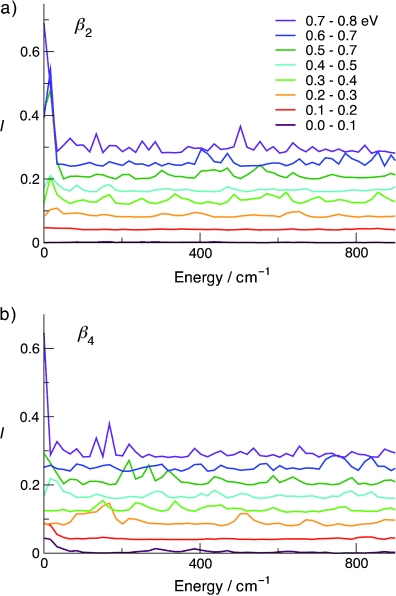
Fourier transforms of photoelectron anisotropy parameters a) *β*_2_(*t*,*E*) and b) *β*_4_(*t*,*E*) obtained by using 0.1 eV energy bins. Curves determined for different photoelectron kinetic energies are shown in different colors and are shifted +0.04*n* (*n*=1–7) vertically for clarity.

For the time evolution of the photoelectron angular anisotropies β_*n*_(*t, E*) (Figure 3 b and c), we were unable to identify a clear time evolution and their Fourier transforms appear to be very flat (Figure 7). This result differs from Bisgaard et al. who observed oscillations in *β_n_*(*t*, *E*) as a function of time[8] by employing a pump laser with a narrower bandwidth (250 cm^−1^) than ours (1740 cm^−1^).

## Discussion

### Decay Profile

The decay profile of the photoionization signal can be well expressed by a single exponential function corresponding to a process shown in Equation (2):


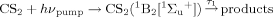
(2)

The addition of an induction time prior to the decay slightly improved the agreement with experimental results. The present result clearly differs from those obtained by Townsend et al. and Bisgaard et al. who proposed that two parallel decay processes occurred with short and long time constants.[7, 8] The short time constant they observed (50–70 fs) was much longer than the time resolution of the present experiment. The absence of the fast component in our results may be due to wave packets created by the broad-band (1740 cm^−1^) excitation propagating in different regions on the potential energy surface of CS_2_ from wave packets prepared by narrow-band excitation.

### Quantum Beats

**Figure 8:**
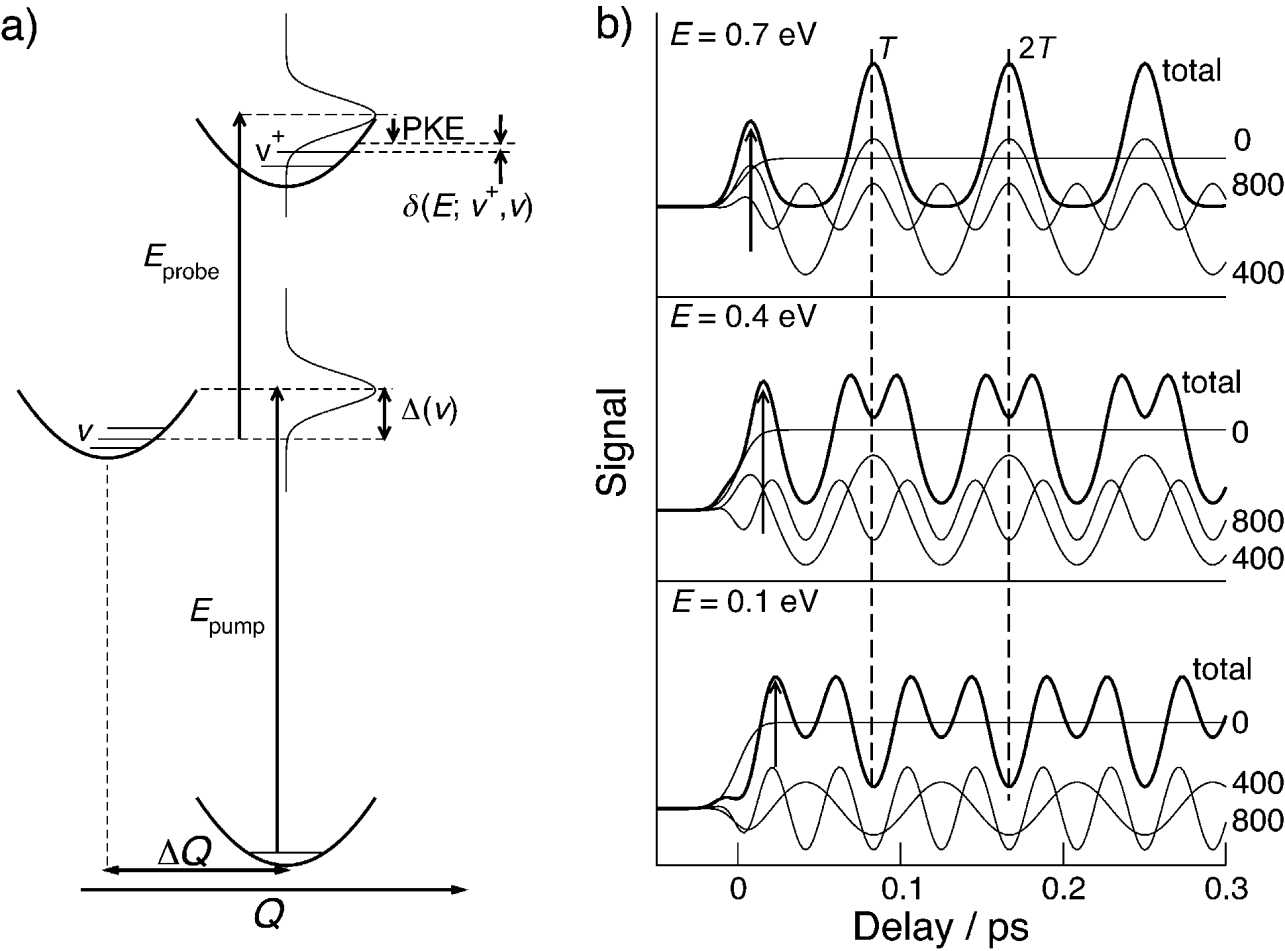
a) Model used to calculate PKED. b) Simulated energy-resolved time profiles of photoelectron signals (black lines) at PKE=0.1, 0.4, and 0.7 eV using a one-dimensional harmonic potential with a fundamental vibrational energy of 400 cm^−1^. The excited-state potential was shifted by Δ*Q=*4. Thin lines indicate relevant beating components of 0, 400, and 800 cm^−1^. The arrows indicate the initial peaks, whereas the dashed lines indicate the vibrational revival times, *T* and 2*T*, in which *T*=*h*/*F*=83 fs; *h* is Plank’s constant, and *F*=400 cm^−1^.

To understand the observed time dependence, we consider a model using Gaussian-shaped pump and probe pulses. The photoelectron signal, σ(*t, E*), is expressed by Equation (3):


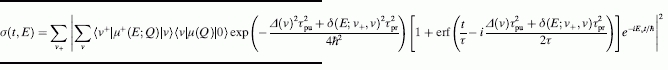
(3)

in which *μ*(*Q*) and *μ*_+_(*E*;*Q*) are the electric transition dipole moments for the pump and probe processes, respectively. *ν* and *ν*_+_ are the vibrational quantum number of the excited state and cation, respectively. |0>, |*ν*>, and |*ν*_+_> denote the vibrational states for the ground and excited states, and cation, respectively. Δ(ν) and δ (*E*;ν_+_, ν) are given by Δ (ν) = *E*_pump_ − *E*_ν_ − *E*_00_ and δ (*E*;ν_+_, ν) = *E*_probe_ + *E*_ν_ + *E*_00_ − *E* − IE − *E*_ν+_, respectively, in which *E*_pump_=*h*ν_pump_ and *E*_probe_=*h*ν_probe_ (Figure 8 a), and *E*_ν_ and *E*_ν+_ are the vibrational energies of the excited state and cation, respectively. *E*_00_ is the excitation energy of the 0–0 band; IE is the ionization energy of CS_2_; τ_pu_ and τ_pr_ are related to the FWHM of the pump and probe pulses by 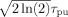 and 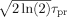, respectively; and erf(*x*+*iy*) is an error function with a complex argument, the limit of which is 1 when *x*≫1. Except for the last one (*e*^−^*iE*_ν_*t*/ħ), all of the factors are real at 
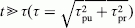
, so they can be transformed into a sum of cos[*E*_ν_ − *E*_ν^′^_)*t*/ħ] functions. Comparison with the damped oscillation function, exp(−*t*/*τ_n_*)cos(2π*f_n_t*+*φ_n_*), gives *φ_n_*=0 or π. Although we neglected molecular rotational degrees of freedom and photoelectron continuum states (i.e., partial waves) in Equation (3), inclusion of these do not alter the phase.

The equilibrium structures of the ground states of both CS_2_ and CS_2_^+^ are linear,[11, 12] whereas the ^1^B_2_ state has a bent equilibrium structure. The absorption spectrum exhibits progressions of the bending and symmetric stretching modes, as seen in Figure 1.[1] The asymmetric stretching mode (1567 cm^−1^) was observed by two-photon absorption spectroscopy,[13] which suggested that ^1^B_2_ was at least quasi-bound upto the (0,0,1) level. However, the same band was not observed by one-photon absorption spectroscopy. We thus neglect the asymmetric stretching mode and consider one- and two-dimensional models to analyze the observed quantum beats. The initially created wave packet at time zero has a dominant Franck–Condon overlap with the (0,0,0) state of the cation, resulted in a high PKE on ionization. The wave packet then moves toward the minimum of the excited-state potential and arrives at the other turning point at a vibrational half-revival time, where ionization creates a vibrationally excited cation and a low-energy photoelectron. Figure 8 b shows a simulation using a one-dimensional harmonic potential with a level spacing of 400 cm^−1^. Because no information was available for the magnitudes of the actual transition dipole moments of CS_2_, we assumed that they were unity, *μ*_+_(*E*;*Q*)=*μ*(*Q*)=1. The initial state was the vibrational ground state in the X state, whereas vibrational states up to *v*=20 of the excited and cation states were included in the calculation. The three most important components (0, 400, and 800 cm^−1^) are shown in Figure 8 b: the phases of the 400 and 800 cm^−1^ components become π at a low PKE (0.1 eV). On the other hand, the experimentally determined phase of the 400 cm^−1^ components was 0 at *E*=0.1 eV, which was similar to the case of *E*=0.4 eV given in Figure 8 b.

As indicated by the arrows in Figure 8 b, the first peak appears at a systematically later time for a lower PKE; the same phenomenon was observed in Figure 4 b. This is the signal from the wave packet moving from the Franck–Condon region toward the outer turning point. The second peak at *E*=0.1 and 0.4 eV in the simulation is due to wave packets reflected from the outer turning point. Thus, the doublet structures at 0.1 and 0.4 eV are due to wave packets moving in opposite directions. This doublet is not observed at *E*=0.7 eV because the molecules are ionized at the turning point. The experimentally observed PKED exhibits similar doublet structures with very small splittings because ionization occurred only in the vicinity of the inner turning point in the Franck–Condon region; the other turning point could not be observed at this probe wavelength. As shown in Figure 8 b, the doublets are formed by the superposition of the 400 and 800 cm^−1^ components with opposite phases (0 and π). This phase difference is clearly seen at *E*<0.4 eV in the observed phase (Figure 6). From Equation (3), the signal from the invisible outer turning point is predicted to be located at *E*=−0.2 eV. If we use a probe pulse with a photon energy that is about 0.2 eV higher, we should be able to observe all of the wave packet motions by photoelectron imaging. The vibrational wave packet motion and the corresponding PKED are simulated in Figure S2 a and b in the Supporting Information; the latter is quite similar to the observed PKED map. One-dimensional wave packet motion is well understood for diatomic molecules such as Na_2_[14–16] and NaI.[17–19]

**Figure 9:**
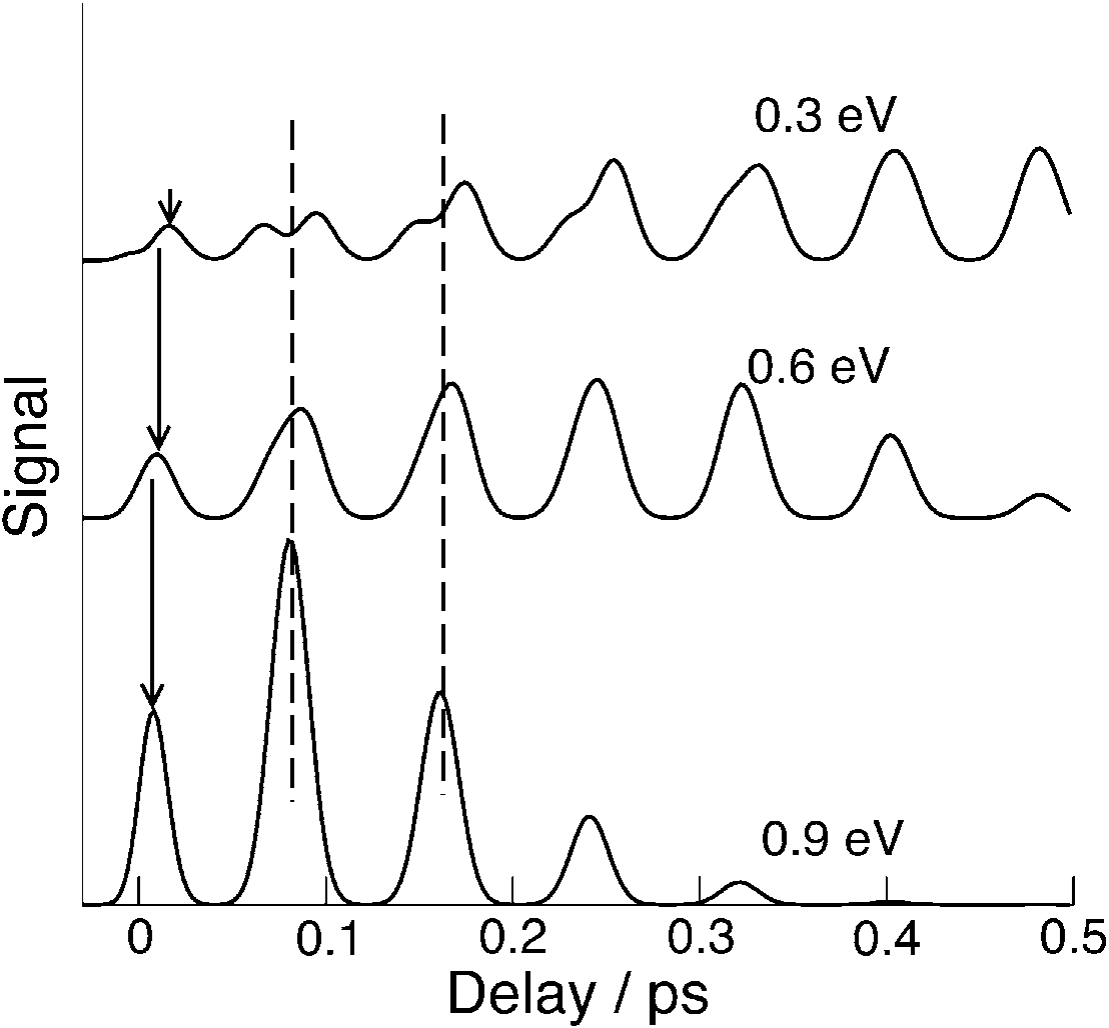
Calculated energy-resolved time profiles of photoelectron signals obtained by using two-dimensional harmonic potentials. The arrows indicate the initial peaks and the dashed lines indicate approximate vibrational revival times.

We also considered the two-dimensional harmonic potential for bending and symmetric stretching modes with experimental[1] harmonic frequencies. Essentially the same features were observed as those for the one-dimensional simulation. Figure 9 shows the results obtained by using displacement parameters of Δ*Q*_sym_=3.2 and Δ*Q*_bent_=4.2, which roughly correspond to the equilibrium geometry[2] of 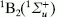, C–S=1.66 Å and ∠S–C–S=153°. The first peaks (denoted by arrows) exhibit continuous delay of the appearance for smaller PKE, while the second and third peaks (denoted by dashed lines) become a doublet or flattened peak at very low PKE. These behaviors agree with the 1D model and the experimental results. Interestingly, the signal at 0.9 eV exhibited a decay, even though we have not included any predissociation in this simulation. This decay is solely due to a slow quantum beat (34 cm^−1^). The slow quantum beat is also operative in the actual case shown in Figure 4 a, as the signal at 0.8 eV becomes almost zero at about 0.4 ps even though the decay constant is approximately 0.5 ps. Thus, the photoelectron signal primarily decays due to predissociation, while a faster disappearance of the high PKE signal is ascribed in part to slow quantum beat.

To more accurately reproduce the observed PKED, it is necessary to consider the asymmetric stretching mode and other electronic states (singlet and triplet).

### PAD

**Figure 10:**
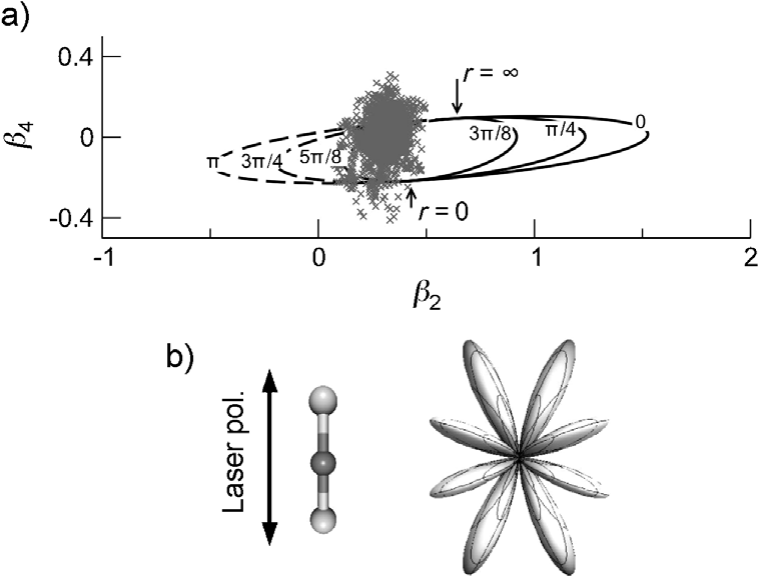
a) *β*_2_–*β*_4_ plot using the two-channel model with relative phases *φ*=0, π/4, 3π/8, 5π/8, 3π/4, and π radians. The range of relative amplitude is 0<*r*<∞. The gray crosses indicate the observed values with standard errors of the mean less than 0.1. b) Molecular frame photoelectron angular distribution obtained using a relative amplitude of *r*=1.8 and a phase of *φ*=1.9 radian.

Figure 10 shows *β*_2_ and *β*_4_ observed (gray crosses) at various time delays and PKEs (0<*t*<2 ps and 0<PKE<1 eV). The values are almost constant at *β*_2_=0.3 and *β*_4_=0.0. We now consider these anisotropy parameters based on the photoelectron partial waves. Partial wave analysis is simple at low PKE because the centrifugal barrier prevents the contribution of high-angular-momentum waves.[20] The ^1^B_2_(^1^Σ_u_^+^; π_u_*←π_g_) state has a quasi-linear structure at >49 000 cm^−1^ (Figure 1),[1] whereas the CS_2_^+^(*X*;^2^Π_g_) state is linear. Therefore, we consider herein that the photoionization dynamics are in a linear geometry. For ionization from the π_u_* orbital, the optically accessible continua are kσ_g_, kπ_g_, and kδ_g_. Bisgaard et al. suggested that the parallel transition (kπ_g_←π_u_*) had a higher intensity than the perpendicular transition (kσ_g_, kδ_g_←π_u_*).[8] We speculate that this is due to the influence of the shape resonance in the kπ_g_ continuum at PKE=4–5 eV.[21–23] If we assume that the maximum angular momentum of the photoelectron is five (*h* wave), there are only two possible channels: dπ_g_ and gπ_g_. This assumption is consistent with the fact that no photoelectron anisotropy higher than *β*_8_ was observed for a highly aligned ensemble of CS_2_.[8] Thus, neglecting the kσ_g_ and kδ_g_ channels, which have small transition dipole moments, the photoelectron anisotropy parameters are determined by the relative phase and amplitude between gπ_g_ and dπ_g_. By using a previously reported general formula,[20] we obtain Equations (4) and (5):


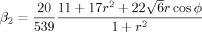
(4)


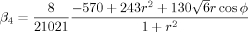
(5)

Equations (4) and (5) account for the molecular alignment created on excitation to 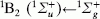. The lines in Figure 10 a indicate *β*_2_ and *β*_4_ calculated for various relative phases and amplitudes. If we use *β*_2_=0.3 and *β*_4_=0.0, we obtain *r*=1.8 and *φ*=±1.9 radians, which results in the molecular frame PAD[24] shown in Figure 10 b: however, our estimate of *r* is crude because of the narrow range of *β*_4_ (−0.23 ≤ β_4_ ≤ 0.10), which corresponds to a very wide range for *r* (0 ≤ *r* ≤ ∞). The sign of phase, *φ*, can be determined from the energy dependence of *β*_2_ or *β*_4_ because their energy dependence at low energy is dominated by the Coulomb phase shift.[24] The subtle decrease of both *β*_2_ and *β*_4_ with increasing PKE suggests that the phase is likely to be +1.9.

## Conclusion

We studied the excited-state dynamics of CS_2_ by photoelectron imaging with a time resolution of 22 fs. Ultrafast, multidimensional bending and stretching dynamics of CS_2_ were observed in which the characteristics of the vibrational quantum beats were consistent with the absorption spectrum. The observed population decay rate was in reasonable agreement with the results of previous studies,[1, 6, 7] while a fast decaying component was not observed in our experiment. The population decay could be approximated by a single exponential with a time constant of about 400 fs, while a small lag time for the decay provided nonexponential behavior. We regard this induction time to be the time for the vibrational wave packet to move from the Franck–Condon region to the critical configuration for predissociation. Analysis of the quantum beats revealed that the wave packets moved in opposite directions. Comparison with a simple theoretical model indicates that the observation window of our photoelectron imaging is limited to the vicinity of the Franck–Condon region at the present probe photon energy. We launched a spatially confined wave packet on the ^1^B_2_(^1^Σ_u_^+^) potential energy surface and examined the photoelectron angular anisotropy to probe the change in the local electronic character of the nonstationary state, but no appreciable variation in PAD was identified. The observed photoelectron angular anisotropies were well explained by the two-channel model (dπ_g_ and gπ_g_).

## Experimental Section

### Time-Resolved Photoelectron Imaging

The apparatus has been described in detail elsewhere.[25] The pump (200 nm) and probe (260 nm) pulses were generated by using a multicolor filamentation light source[26, 27] based on a cryogenically cooled Ti:sapphire amplifier (pulse energy: 2 mJ; pulse length: 25 fs; wavelength: ≈780 nm; repetition rate: 1 kHz). The deep-UV pulses from the filamentation cell were compressed by grating compressors. Their pulse widths were 14 (260) and 17 fs (200 nm). The delay time (*t*) between them was controlled by using a closed-loop translation stage. The pump and probe pulses were focused by a concave mirror (*r*=1500 mm) onto a supersonic molecular beam of carbon disulfide (≈7 %) seeded in helium carrier gas (stagnation pressure: 760 torr). The intersection angle between the pump and probe pulses was ≈0.8. To prevent one-color multiphoton processes, the pump and probe pulse energies were reduced to ≈10 (200) and 200 nJ per pulse (260 nm), respectively, by variable apertures.

Photoelectrons generated by (1+1′) resonance-enhanced multiphoton ionization were accelerated in the molecular-beam propagation direction and projected onto a two-dimensional position-sensitive detector consisting of a dual microchannel plate (75 mm*φ*), a phosphor screen, and an image-intensified charge-coupled device camera (1024×1024 pixels). The polarization directions of the pump and probe beams were aligned parallel to each other and parallel to the face of the microchannel plate detector. Images were obtained for 13.3 fs intervals in the delay time and the acquisition time for a single scan at each delay time was 4 s. 150 images were successively obtained in a single scan from −87 to 1900 fs and 10 scans were performed. The three-dimensional photoelectron velocity and angular distributions were reconstructed from the observed projection images by using the pBaseX method.[28]

The PKE was calibrated by observing one-color three-photon ionization of xenon at 260 nm. The broad spectra of the pump and probe pulses limited the energy resolution of the present photoelectron imaging system to about 0.3 eV (FWHM). The cross-correlation of the pump and probe pulses was confirmed in situ to be 22 fs by nonresonant (1+1′) multiphoton ionization of ethanol seeded in a supersonic jet of argon. The measured time profiles of the electron and CS_2_^+^ signals are in excellent agreement (Figure S3 in the Supporting Information), which is consistent with cluster and fragment ions not being detected in the TOF mass spectra.

### Fitting Procedure

It is generally difficult to fit a transient signal with damped oscillations by nonlinear least-squares fitting. One of the most effective methods is the combination of linear prediction and linear least-squares fitting. Therefore, we performed the fitting as follows: 1) We extracted frequencies (*f_n_*; *n*=2,3,4,5) and damping times (*τ_n_*; *n*=2,3,4,5) of the oscillations from the total photoelectron signal using linear predictive coding (including the overall signal decay time (*τ*_1_) without oscillations). 2) We obtained the overtone frequencies (*f*_6_ and *f*_7_) and decay time constants (*τ*_6_ and *τ*_7_) from the photoelectron signal at 0.7 eV. 3) Linear least-squares fitting was performed, including damped oscillations and the apparatus function (Gaussian with a FWHM of 22 fs). The frequencies, *f_n_*, and time constants, *τ_n_*, were constant over the entire PKE region, whereas the amplitude, *A_n_*(*E*), and phase, *φ_n_*(*E*), of the oscillations were determined at each PKE. We added decay, exp(−*t/τ*′), and rise, −exp(−*t/τ*′), components to imitate nonexponential decay; the time constant, *τ*′, is regarded as the induction time. 4) We minimized the residuals, *χ*^2^, by varying the induction time. The obtained induction time was 28 fs. Thus, the basis function for the linear least-squares fitting was the convolution between the apparatus function and response function given by Equation (6):


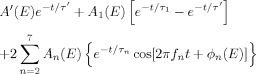
(6)

Figures 2, 4, and 6 show the results of the fitting and Table 1 lists the common parameters. The four oscillatory components (*hf_n_*=34, 350, 394, 416 cm^−1^) extracted by the above method were reasonably similar to those obtained by taking the Fourier transform (40, 338, 386, 424 cm^−1^) of the total photoelectron intensity signal (Figure 2 a). The amplitude *A*′(*E*), which corresponded to the initial ^1^B_2_ population, was slightly negative at some energies probably owing to deficiencies in the kinetic model expressed by Equation (6); convolution of Equation (6) with the apparatus function gave a linear combination of error functions, whereas there were squared error functions in the quantum-mechanical formula of Equation (3). The lifetime (*τ*_1_=470 fs) agreed reasonably well with those found by previous time-domain experiments (400–600 fs) at a similar pump wavelength.[6, 7] The induction time (28 fs) was similar to the short time constant (≈50 fs) previously obtained by a parallel decay model.[7, 8] We examined the parallel decay model using our data; however, it provided unacceptable negative intensities at all energies for the rapidly decaying component.

**Table 1:** Parameters for the basis functions

	*hf* [cm^−1^]		*τ* [fs]
–	–	*τ*′	27.5
–	–	*τ*_1_	465
*f*_2_	34	*τ*_2_	309
*f*_3_	350	*τ*_3_	450
*f*_4_	394	*τ*_4_	361
*f*_5_	416	*τ*_5_	250
*f*_6_	768	*τ*_6_	568
*f*_7_	818	*τ*_7_	360
