# Supplementary material for: Excited-State Dynamics of CS2 Studied by Photoelectron Imaging with a Time Resolution of 22 fs
Source: Chem Asian J. 2011 Oct 13;6(11):3028–34. doi: 10.1002/asia.201100458 (PMC3263315; doi:10.1002/asia.201100458)
Supplement: Supplementary file 1 [file asia0006-3028-SD1.pdf]

CHEMISTRY
AN ASIAN JOURNAL

Supporting Information

© Copyright Wiley-VCH Verlag GmbH & Co. KGaA, 69451 Weinheim, 2011

Excited-State Dynamics of CS_2 Studied by Photoelectron Imaging with a Time Resolution of 22 fs

Takao Fuji,[a, b] Yoshi-Ichi Suzuki,[a, b, c] Takuya Horio,[a, b, c] and Toshinori Suzuki*[a, b, c]

asia_201100458_sm_miscellaneous_information.pdf

Supporting information

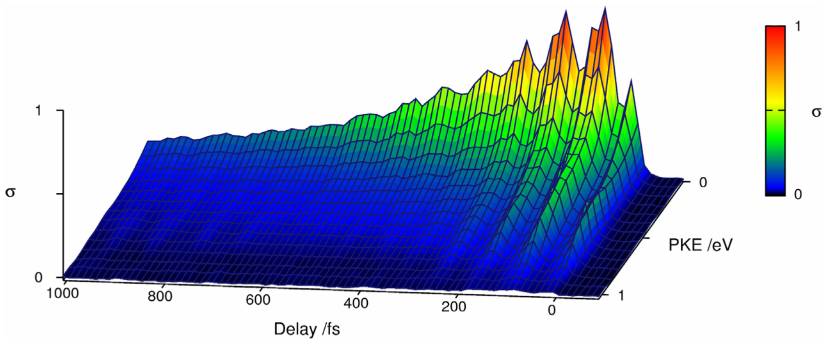

Fig. S1 Time evolution of photoelectron intensities as a function of PKE and pump-probe delay.

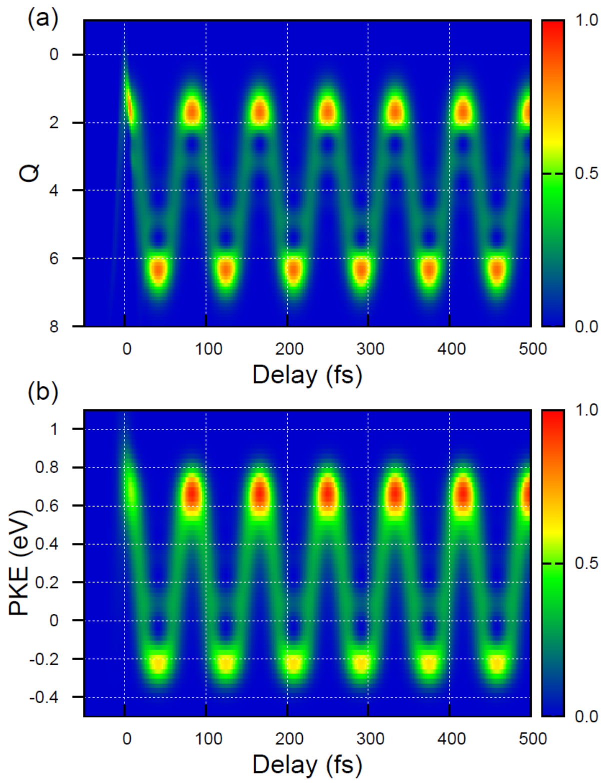

Fig. S2 (a) Time evolution of a wave packet on a single harmonic potential (see Fig. 8), and (b) corresponding photoelectron kinetic energy distribution as a function of pump-probe delay. Using Eq. (3), the photoelectron intensities were calculated also at negative energy to compare them with the wave packets, although these signals cannot be observed experimentally.

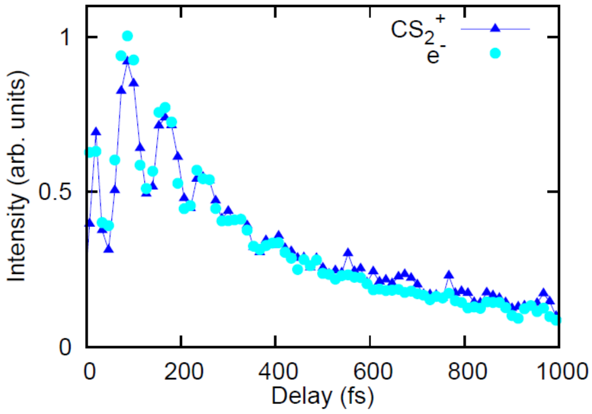

Fig. S3 Electron and ion signals upon (1+1') resonant photoionization of CS_2.
